# Real-world evidence of epidemiology, patient characteristics, and mortality in people with drug-resistant epilepsy in the United Kingdom, 2011–2021

**DOI:** 10.1007/s00415-023-12165-4

**Published:** 2024-01-19

**Authors:** Rohit Shankar, Xiaocong Li Marston, Vanessa Danielson, Bronwyn Do Rego, Reginald Lasagne, Oliver Williams, Lara Groves

**Affiliations:** 1 Peninsula School of Medicine, University of Plymouth, Plymouth, PL4 8AA UK; 2 OPEN Health Evidence and Access, 20 Old Bailey, London, EC4M 7AN UK; 3 LivaNova, Gloucester, UK

**Keywords:** Real-world evidence, Refractory epilepsy, Epidemiology, Anti-seizure medications, Disease burden, Intellectual disabilities

## Abstract

**Background:**

A third of people with epilepsy are drug resistant. People with drug-resistant epilepsy (DRE) have a higher risk of mortality and physical injuries than those who respond to anti-seizure medication (ASM). This study describes patient characteristics, comorbidities, and mortality in people with DRE in the UK.

**Methods:**

The Clinical Practice Research Datalink was utilised to select people with DRE prescribed a third ASM between 1 January 2011 and 31 March 2021. Annual incidence and prevalence of DRE, patient characteristics, comorbidities, and mortality rates were analysed. Subgroup analysis was performed by age, sex, presence of intellectual disabilities and time from epilepsy diagnosis to DRE.

**Results:**

A total of 34,647 people with DRE were included (mean ± SD age 42.68 ± 23.59 years, 52.6% females). During the study period, annual DRE incidence ranged from 1.99% to 3.12%. As of 31 March 2021, DRE prevalence was 26.6% (95% confidence interval [CI] 26.3%–26.8%). A greater proportion of people with DRE resided in the most deprived regions, with 21.1% and 16.7% in the top two quintiles of the Index of Multiple Deprivation respectively, compared to < 15% in the three less deprived regions. All-cause mortality ranged from 3,687 to 4,802 per 100,000 persons with DRE, four times higher than that in the general population in the UK. Variations existed across subgroups.

**Conclusions:**

Considerable disease burden was observed in people with DRE in the UK. The findings emphasise the importance of early DRE diagnosis and appropriate disease management in people who develop DRE.

**Supplementary Information:**

The online version contains supplementary material available at 10.1007/s00415-023-12165-4.

## Introduction

People with epilepsy can experience recurrent epileptic seizures without any immediately identifiable cause [1]. Currently, anti-seizure medications (ASMs) are the mainstay of treatment. However, approximately a third of people with epilepsy have drug-resistant epilepsy (DRE) [2, 3]. The definition of DRE proposed by the International League against Epilepsy (ILAE) in 2010 has been internationally accepted: people with epilepsy experiencing a “failure of adequate trials of two well-tolerated and appropriately chosen and used ASM schedules (whether as monotherapies or in combination) to achieve sustained seizure freedom [4].”

People with DRE may achieve control of their symptoms with the third drug or remain uncontrolled and continue to experience seizures which can severely affect their quality of life and increase caregivers’ burden [4]. People with DRE have been shown to experience a higher risk of mortality and physical injuries compared to people with controlled epilepsy [5]. People with DRE also incur increased resource utilisation and pose a greater burden to healthcare systems [6, 7]. It has been reported that the healthcare needs of people with DRE differ depending on age, sex, disease duration, and presence of intellectual disabilities [8, 9]. However, most studies regarding DRE and patient profiles have been performed in the United States (US), and there is limited information on the current burden of DRE in the United Kingdom (UK) and the characteristics of this patient population.

An understanding of people with DRE and the associated impact on mortality and morbidity would be useful to better characterise the unmet need of this patient population and to improve the management of care for those people within the National Health Service (NHS). The current study was conducted to quantify DRE as defined by the ILAE criteria in the UK and to describe the patient characteristics [4], comorbidities, and mortality of this patient population. The goal of the study was to provide up-to-date epidemiological evidence on people with DRE in the UK. The findings of the study may be used by clinicians, researchers, and policymakers to better characterise the unmet need of this patient population and help plan patient pathways and services for people with epilepsy and their carers.

## Methods

This is a retrospective, non-interventional cohort study using data from the Clinical Practice Research Datalink (CPRD) databases from 1 January 2011 to 31 March 2021.

### Data source

CPRD is a real-world research UK-based service supporting retrospective and prospective public health and clinical studies. CPRD is jointly sponsored by the Medicines and Healthcare Products Regulatory Agency (MHRA) and the National Institute for Health and Care Research (NIHR), as part of the Department of Health and Social Care. CPRD collects de-identified primary care data from a network of general practices across the UK to provide a longitudinal, representative UK population health dataset [10]. The protocol of the current study (Protocol Reference ID: 22_002201) was approved by the data governance framework for CPRD, underpinned by the Research Data Governance (RDG) process.

Data on patient demographics, such as age, sex, and geographical location; comorbid conditions; and prescriptions are available from CPRD. Linked data from the Office for National Statistics (ONS) were used to provide information on patient death status and cause of death.

### Study design and study sample

**Fig. 1:**
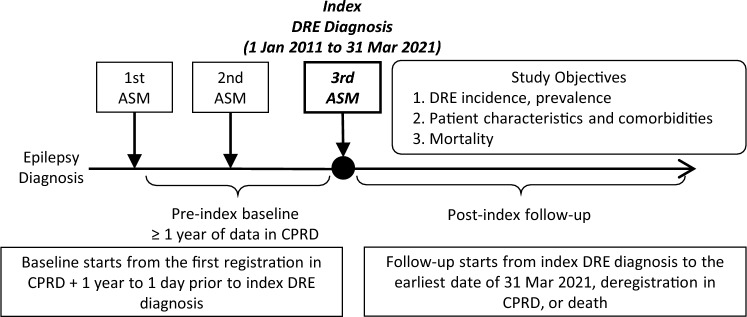
Schematic Diagram. Abbreviations: *ASM* anti-seizure medication, *CPRD* Clinical Practice Research Datalink, *DRE* drug-resistant epilepsy

Figure 1 depicts the study design. The 2010 ILAE criteria were used as a proxy to define the DRE population because no DRE-specific clinical codes are available [4]. Based on the ILAE criteria [4], DRE was defined if a person with epilepsy had a third ASM prescribed as a monotherapy or in combination. Read codes and Systematized Nomenclature of Medicine (SNOMED) codes recorded in the CPRD databases were used to search epilepsy diagnosis (see Online Resource 1 for diagnosis codes) from patients’ date of first registration plus one year until the earliest of 31 March 2021, date of deregistration in CPRD (e.g., due to relocation), or date of death. Then, prescription data from 1 January 2011 through 31 March 2021 in CPRD were extracted amongst people with epilepsy to define people with DRE who had a third ASM prescribed (see Online Resource 2 for prescription codes).

The index date was defined as the date of DRE diagnosis, which was the earliest date within the observational period (1 January 2011 through 31 March 2021) that the third unique ASM was prescribed for a person with epilepsy. The baseline period started at a patient’s first registration into CPRD plus one year and ended on the day prior to the index date. A minimum of one year of data in CPRD during the baseline period was required for a patient to be included in the study sample to observe baseline characteristics and comorbidities. The follow-up period started on the index date and ended on the earliest of 31 March 2021, date of deregistration in CPRD, or date of death.

### Study objectives and variables

The study has the following objectives:

1. To quantify DRE as defined by the ILAE criteria in the UK.
2. To describe patient characteristics and comorbidities for people with DRE in the UK.
3. To assess all-cause mortality and epilepsy-related mortality among people with DRE in the UK.

Patient characteristics and comorbidities were assessed at the time of the index date and during the baseline period. Mortality occurring any time after the index date was reported. Summaries of the study variables and the operational definitions can be found in **Online Resource 3**. The Wirrell et al. (2022) approach was adopted to define the type of epilepsy using a coded entry in CPRD from the first epilepsy diagnosis up to the day prior to the index date [11]. Four different types of epilepsy were defined according to Wirrell et al. [11]: generalised epilepsy, focal epilepsy, developmental and epileptic encephalopathy (DEE), and unclassifiable epilepsy (see Online Resource 1 for categorisation). A person may have multiple diagnoses of epilepsy recorded in CPRD from the first epilepsy diagnosis up to the day prior to the index date. Each of these diagnoses may meet the definition of one of the four types of epilepsy. Therefore, a person could have more than one type of epilepsy recorded in CPRD. The following types of epilepsy and combinations of records were reported: generalised epilepsy, focal epilepsy, DEE, focal and generalised epilepsy, generalised epilepsy and DEE, focal epilepsy and DEE, focal / generalised epilepsy and DEE, and unclassifiable epilepsy.

### Statistical analysis

All analyses in this study were descriptive. Summary statistics were presented as frequencies and percentages for categorical variables and as means, standard deviations (SDs), medians, interquartile ranges (IQRs), minimum, and maximum for continuous variables. Subgroup analysis was conducted by sex (males vs. females); age (children aged < 18 years old vs. adults aged ≥ 18 years old at index); intellectual disabilities (people with vs. without a diagnosis of intellectual disabilities; see Online Resource 4 for code list); time from epilepsy diagnosis (patients with time from the first epilepsy diagnosis on record to the index DRE diagnosis < median vs. ≥ median). No statistical comparisons were performed between subgroups.

Patients with missing data on the Index of Multiple Deprivation (IMD) were categorised as “unknown”; no other study variables had missing data. Small-number suppression was performed in line with CPRD guidance [12]. To prevent the risk of unintentional disclosure of patients, all numbers with < 5 events or patients were suppressed in figures, tables, and text. All analyses were performed using R Statistical Software (version 4.2.2) and Microsoft SQL.

## Results

### Study sample

**Fig. 2:**
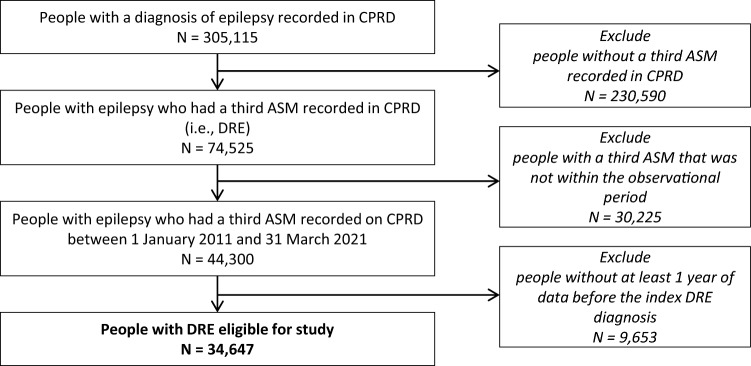
Flowchart of Patient Selection. Abbreviations: *ASM* anti-seizure medication, *CPRD* Clinical Practice Research Database, *DRE* drug-resistant epilepsy

A total of 305,115 people had at least one diagnosis of epilepsy recorded in CPRD. Of these patients, 34,647 people with DRE were eligible for analysis. Figure 2 shows the flow chart of the patient selection process.

### DRE incidence and prevalence in the United Kingdom

**Fig. 3:**
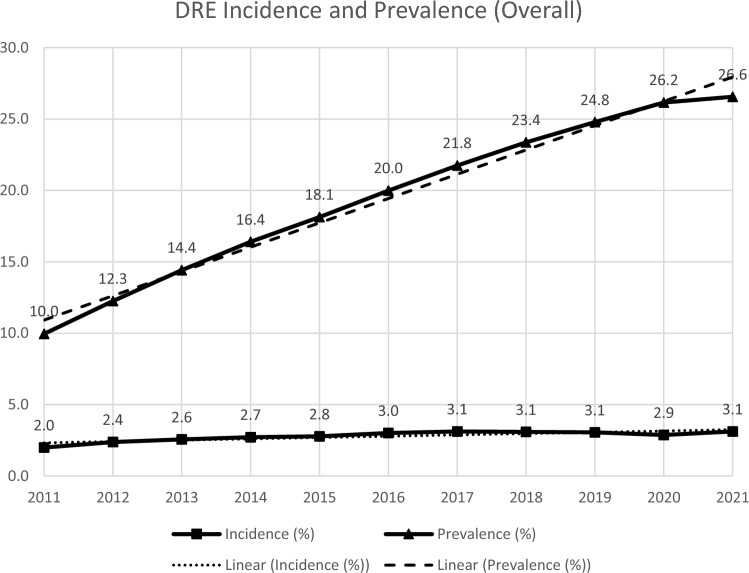
Annual DRE Incidence and Prevalence in People with Epilepsy, 2011 – 2021. Abbreviations: *DRE* drug-resistant epilepsy. Note: A linear fitted trendline is shown in the dashed line for incidence and prevalence, respectively. For the last year when only partial data of the year were available (i.e., through March 2021), the incidence of the year was annualised by multiplying the number of DRE cases in the first three months of 2021 by four; the prevalence of the year was measured on 31 March 2021. For all other years, incidence was measured using the actual number of cases and prevalence was measured on 31 December of the year

The annual incidence of DRE ranged from 1.99% in 2011 to 3.12% in 2021 among individuals with epilepsy (Fig. 3). The accumulated prevalence of DRE increased from 10.0% in 2011 to 26.6% in 2021 among individuals with epilepsy (Fig. 3).

### Characteristics of people with DRE

#### Patient demographics

The average age at the time of DRE diagnosis was 42.68 ± 23.59 years (mean ± SD) in the study population, with 17.7% being children (< 18 years) and 82.3% being adults. Adult patients were evenly distributed across different age groups (Table 1). Females accounted for 52.6% of all people with DRE. A disproportionate number of people with DRE were seen in the most deprived regions that were in the top two quintiles (21.1% and 16.7% in quintile 1 and quintile 2, respectively) of the IMD compared to regions that were in the three less deprived quintiles (14.8%, 13.8%, and 12.3% for quintile 3, 4, and 5, respectively).

**Table 1:** Characteristics of People with DRE

Variable	Statistic	All patients *N* = 34,647
Age at index (time of the initiation of the third recorded ASM prescription), year	Mean (SD)	42.68 (23.59)
	Median (Q1, Q3)	43.00 (23.00, 61.00)
	Min, Max	1.00, 105.00
Age at index, *N* (%)	< 18 years	6135 (17.7%)
	18 to 30 years	5584 (16.1%)
	31 to 40 years	4450 (12.8%)
	41 to 50 years	5044 (14.6%)
	51 to 60 years	4720 (13.6%)
	61 to 70 years	3748 (10.8%)
	71 years and above	4966 (14.3%)
Sex, *N* (%)	Female	18,239 (52.6%)
	Male	16,408 (47.4%)
Index of multiple deprivation, *N* (%)	Q1 Most deprived	7308 (21.1%)
	Q2	5798 (16.7%)
	Q3	5137 (14.8%)
	Q4	4793 (13.8%)
	Q5 Least deprived	4247 (12.3%)
	Unknown	7364 (21.3%)

*95% CI* 95% confidence interval, *ASM* anti-seizure medication, *DRE* drug-resistant epilepsy, *Median (Q1, Q3)* median (first, third quartile), *NA* not applicable, *Q1 to Q5 in Index of Multiple Deprivation* quintile 1 to quintile 5, *N* number, *SD* standard deviation

**Fig. 4:**
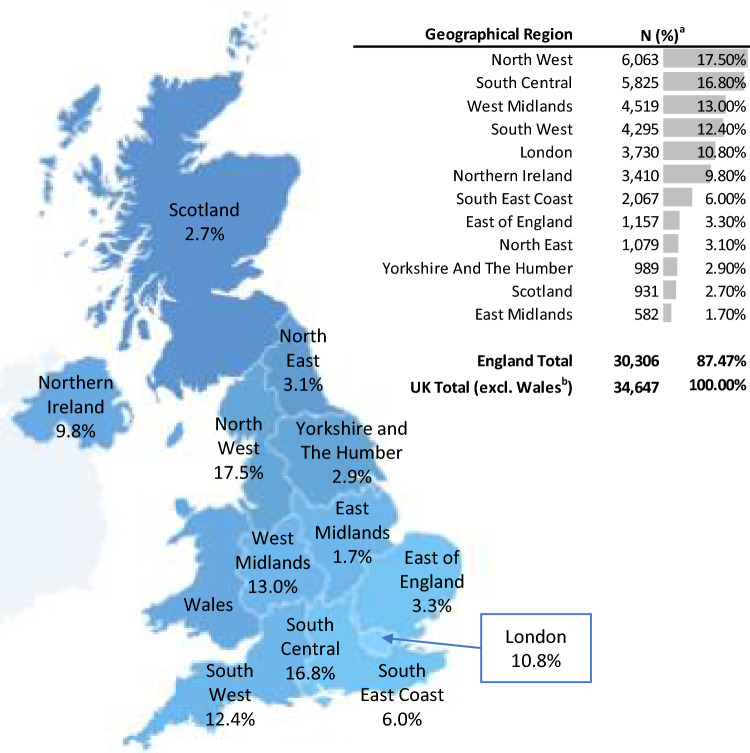
Geographical distribution of people with DRE in the United Kingdom. Abbreviations: *DRE* drug-resistant epilepsy, *N* number. Notes: ^a^Results reflect the population coverage of Clinical Practice Research Datalink practices, which do not necessarily align with the real geographical distribution of DRE in the UK. Results should be interpreted with caution. ^b^Data from Wales are included in the Clinical Practice Research Datalink but under-represented. No Welsh patients with DRE were included in the study sample

Figure 4 depicts the geographical distribution of people with DRE across the UK. Northwest England (17.5%), the South Central (16.8%), West Midlands (13.0%), Southwest (12.4%) and greater London (10.8%) accounted for the most patients with DRE in the UK, while the East Midlands, Scotland, Yorkshire and the Humber, Northeast England, and East of England each accounted for a small proportion of people with DRE (ranging from 1.7% to 3.3%).

### Comorbidities and epilepsy for people with DRE

The average (mean ± SD) Charlson Comorbidity Index (CCI) score was 1.14 ± 1.62 in people with DRE, ranging from 0 to 14 (Table 2). Approximately half of the study cohort (49.7%) scored zero for the CCI. One-third (34.4%) scored 1–2, while the rest 16.0% scored greater than 2. Among people with DRE, acute bronchitis and bronchiolitis (11.5%) were the most common comorbidity within one year prior to the index date. Other common comorbidities included soft tissue disorders (e.g., shoulder pain and leg pain related to rheumatism, 11.2%), back problems (7.4%), and other acute upper respiratory infections (6.6%).

**Table 2:** Comorbidities and Epilepsy in People with DRE

Variable	Statistic/Category	All patients *N* = 34,647	Adults (Age ≥ 18 years) *N* = 28,512	Children (Age < 18 years) *N* = 6135
*Charlson Comorbidity Index (CCI)*				
	Mean (SD)	1.14 (1.62)	1.30 (1.70)	0.40 (0.79)
	Median (Q1, Q3)	1.00 (0.00, 2.00)	1.00 (0.00, 2.00)	0.00 (0.00, 1.00)
	Min, Max	0.00, 14.00	0.00, 14.00	0.00, 6.00
*Charlson Comorbidity Index (CCI) category, N *(%)				
	0	17,203 (49.7%)	12,633 (44.3%)	4,570 (74.5%)
	1–2	11,916 (34.4%)	10,539 (37.0%)	1,377 (22.4%)
	> 2	5,528 (16.0%)	5,340 (18.7%)	188 (3.1%)
*The top 10 most frequently recorded diagnoses, N (%)*^a^				
Acute bronchitis and bronchiolitis	1	3,971 (11.5%)	–	–
Other soft tissue disorders	2	3,888 (11.2%)	–	–
Back problem	3	2,564 (7.4%)	–	–
Other acute upper respiratory infections	4	2,280 (6.6%)	–	–
Neurotic disorder	5	1,863 (5.4%)	–	–
Other urethral and urinary tract disorders	6	1,789 (5.2%)	–	–
Disorder of external ear	7	1,483 (4.3%)	–	–
Asthma	8	1,441 (4.2%)	–	–
Diabetes mellitus	9	1,382 (4.0%)	–	–
Joint disorder	10	1,340 (3.9%)	–	–
History of intellectual disabilities	*N* (%)	4313 (12.4%)	2870 (10.1%)	1443 (23.5%)
*Type of epilepsy*^b^				
Generalised epilepsy	*N* (%)	5033 (14.5%)	3740 (13.1%)	1293 (21.1%)
Focal epilepsy	*N* (%)	5483 (15.8%)	4650 (16.3%)	833 (13.6%)
Developmental and epileptic encephalopathy (DEE)	*N* (%)	51 (0.1%)	8 (0.0%)	43 (0.7%)
Focal and generalised epilepsy	*N* (%)	842 (2.4%)	684 (2.4%)	158 (2.6%)
Generalised epilepsy and DEE	*N* (%)	9 (0.0%)	5 (0.0%)	< 5
Focal epilepsy and DEE	*N* (%)	7 (0.0%)	< 5	7 (0.1%)
Focal epilepsy, generalised epilepsy, and DEE	*N* (%)	< 5	< 5	< 5
Unclassifiable epilepsy	*N* (%)	23,220 (67.0%)	19,424 (68.1%)	3796 (61.9%)
*Time from epilepsy diagnosis to DRE diagnosis, years*	Mean (SD)	12.43 (14.60)	14.34 (15.34)	3.55 (3.74)
	Median (Q1, Q3)	6.70 (1.55, 18.20)	9.21 (2.11, 21.58)	2.13 (0.80, 5.07)
	Min, Max	0.00, 86.02	0.00, 86.02	0.00, 17.98
*Time from epilepsy diagnosis to DRE diagnosis, years*	< 1 year	1,959 (5.7%)	1,611 (5.7%)	348 (5.7%)
	1	4923 (14.2%)	3461 (12.1%)	1462 (23.8%)
	2	3023 (8.7%)	1889 (6.6%)	1134 (18.5%)
	3	2251 (6.5%)	1523 (5.3%)	728 (11.9%)
	4	1738 (5.0%)	1230 (4.3%)	508 (8.3%)
	5	1487 (4.3%)	1093 (3.8%)	394 (6.4%)
	6	1199 (3.5%)	908 (3.2%)	291 (4.7%)
	7	1086 (3.1%)	844 (3.0%)	242 (3.9%)
	8	1019 (2.9%)	806 (2.8%)	213 (3.5%)
	9	928 (2.7%)	763 (2.7%)	165 (2.7%)
	10	811 (2.3%)	689 (2.4%)	122 (2.0%)
	11	828 (2.4%)	697 (2.4%)	131 (2.1%)
	12	781 (2.3%)	688 (2.4%)	93 (1.5%)
	13	788 (2.3%)	704 (2.5%)	84 (1.4%)
	14	673 (1.9%)	593 (2.1%)	80 (1.3%)
	15	649 (1.9%)	604 (2.1%)	45 (0.7%)
	> 15 years	10,504 (30.3%)	10,409 (36.5%)	95 (1.5%)

*DRE* drug-resistant epilepsy, *Median (Q1, Q3)* median (first, third quartile), *N* number, *SD* standard deviation

Note: ^a^Comorbidities were defined using data within 1 year prior to the index date based on the leading 3 digits of Read codes. ^b^A person may have multiple diagnoses of epilepsy recorded in the database. The combinations of types of epilepsy (e.g., focal and generalised epilepsy) indicate that a person had a record of different types of epilepsy at different time points from the first record of epilepsy up to the day prior to the index DRE diagnosis

Among people with DRE, two-thirds (67.0%) had unclassifiable epilepsy. In the remaining one-third of the DRE cohort, around half had generalised epilepsy (14.5%) and another half had focal epilepsy (15.8%). People with DEE or mixed epilepsy accounted for an insignificant proportion (Table 2). The median time between an epilepsy diagnosis to a DRE diagnosis was 6.7 years.

### Mortality in people with DRE

**Fig. 5:**
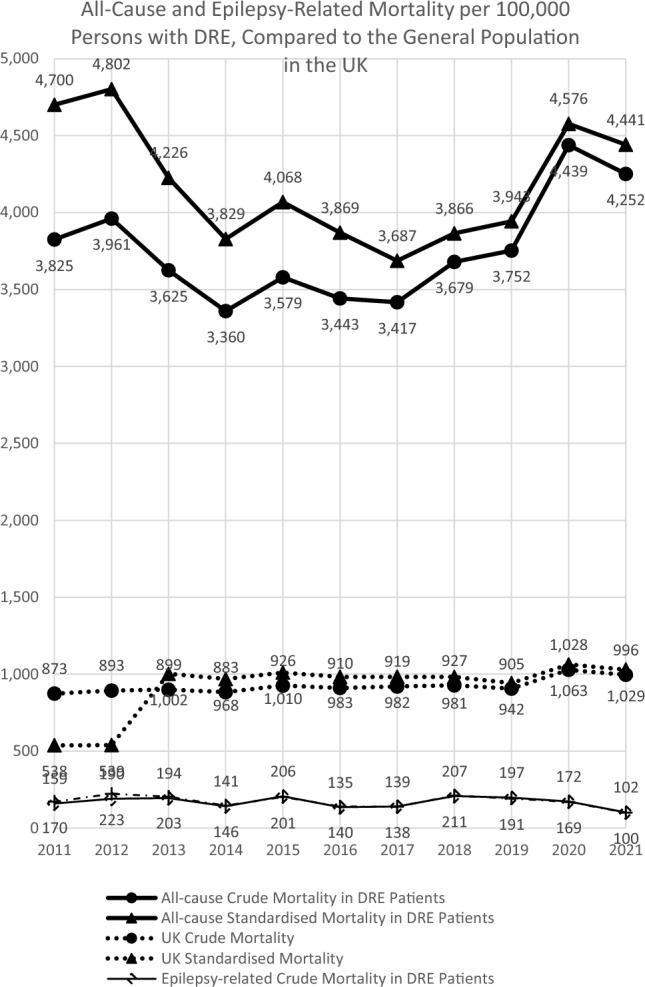
All-cause and Epilepsy-Related Mortality in People with DRE Compared to the All-Cause Mortality in the General Population in the United Kingdom. Abbreviations: *DRE* drug-resistant epilepsy. Notes: ^a^All-cause crude mortality in people with DRE was estimated by dividing the number of all deaths recorded in the Office for National Statistics death registry in a given calendar year by the total number of alive people with DRE on 31 December of the year. ^b^For the last year when only partial data of the year were available (i.e., through March 2021), mortality was estimated on 31 March 2021. ^c^Data on UK crude mortality and standardised mortality were obtained from the Office for National Statistics death registry [13]. ^d^Standardised mortality rate was estimated by dividing the age- and sex-adjusted observed mortality by the age- and sex-adjusted expected mortality in a reference European population. ^e^Epilepsy-related crude mortality was estimated by dividing the number of people with DRE who were deceased with epilepsy as the primary cause of death as recorded in the ONS death registry in a given calendar year by the total number of alive people with DRE on 31 December of the year

The crude all-cause mortality ranged from 3,417 to 4,439 per 100,000 persons with DRE during the period from 2011 to 2021. The age- and sex-standardised mortality during the study period ranged from 3,687 to 4,802 per 100,000 persons with DRE (Fig. 5). Epilepsy-related crude mortality (i.e., people who were deceased with epilepsy as the primary cause of death as recorded in the ONS death registry) ranged from 102 to 207 per 100,000 persons with DRE between 2011 and 2021. When adjusted by age and sex, epilepsy-related standardised mortality ranged from 100 to 223 per 100,000 persons with DRE between 2011 and 2021 (Fig. 5). Overall, 2%-6% of people with DRE died of epilepsy-related causes every year.

### Subgroup analysis

Subgroup analysis was conducted by age (adults vs. children), sex (male vs. female), time from epilepsy to DRE diagnosis (≥ vs. < median), and history of intellectual disabilities (with vs. without intellectual disabilities). The average (mean ± SD) age among children with DRE was 8.98 ± 4.98 years and 49.93 ± 19.34 years among adults. Females with DRE were slightly older than males (43.65 vs. 41.61 years). People with a longer time between epilepsy and DRE diagnosis (≥ 6.7 years) were older (47.95 vs. 37.41 years) than those with a shorter time between epilepsy and DRE diagnosis (< 6.7 years).

People with intellectual disabilities were younger than those without (29.53 vs. 44.55 years). While most subgroups had more females than males, the proportion of males with DRE was higher in children (51.5%) and in those with intellectual disabilities (57.9%). The distribution of geographical location and socialeconomic status as measured by the IMD for people with DRE was similar across different subgroups.

As shown in Table 2, children (mean ± SD: 0.40 ± 0.79) and people with intellectual disabilities (0.61 ± 1.06) scored lower on the CCI compared to the overall DRE cohort (1.14 ± 1.62) and other subgroups (adults 1.30 ± 1.70; without intellectual disabilities 1.21 ± 1.67). Generalised epilepsy was more common in children (21.1% vs. 14.5% overall), people with intellectual disabilities (16.7%), and people with a longer time between epilepsy and the DRE diagnosis (16.1%), whereas focal epilepsy was more common among those with a shorter time between epilepsy and the DRE diagnosis (18.3% vs. 15.8% overall) and those without intellectual disabilities (16.6%). Females had a higher proportion of both generalised (15.4% vs. 13.6%) and focal epilepsy (16.6% vs. 14.9%) compared to their male counterparts. The proportion of DEE was higher in children (0.7% vs. 0.1% overall).

Incidence and prevalence were both higher among children, females, people with a longer time between epilepsy and the DRE diagnosis, and people with intellectual disabilities compared to their counterparts. All-cause mortality (both crude and standardised) was higher in males and adults. All-cause crude mortality was higher in people without intellectual disabilities, whereas standardised all-cause mortality was higher in those with intellectual disabilities. No consistent difference in all-cause mortality (both crude and standardised) was observed between subgroups by time from epilepsy to DRE. The differences in epilepsy-related mortality rates were similar across subgroups.

## Discussions

The results of this study contribute to the knowledge of the disease burden of DRE and patient characteristics in the UK. As of 31 March 2021, the observed prevalence of DRE in the study (26.6%, 95% CI 26.3%–26.8%) was slightly lower than previously reported [2, 3]. In two previous meta-analysis studies, Kalilani et al. reported an overall prevalence of 30% (95% CI 19%–42%) [2], while Sultana reported a prevalence of 13.7% (95% CI 9.2%–19.0%) in population / community-based populations and 36.3% (95% CI 30.4%–42.4%) in clinic-based cohorts [3]. The annual incidence was also lower in the current study (< 5%) compared to the literature (Kalilani 15%, 95% CI 11%–19%; Sultana 19.6%, 95% CI 14.4%–25.4%) [2, 3].

Variations in the reported incidence and prevalence estimates could be attributed to the approach to define DRE, patient inclusion criteria, patient characteristics, study period, and data sources. For example, Kalilani et al. reported that the definition of DRE varied widely across studies, with only 12% meeting the ILAE criteria [2]. Sultana initially excluded studies using the ILAE criteria because it was a newer definition and large-scale population-based studies were not expected to adopt it [3]. Sultana et al. reported that clinic-based studies (vs. population / community-based) and focal epilepsy studies (vs. mixed or generalised epilepsy) were associated with higher prevalence [3].

The point prevalence of epilepsy in the UK was approximately 0.5% in our study using the CPRD database (= 305,115 people with epilepsy divided by the UK population 67,736,802), while the prevalence of epilepsy in England is estimated at 0.9% [14]. Thus, the study captures more than half of the population living with epilepsy in England. It is expected that this comprehensive sample is representative of the full population of epilepsy in England. Further, CPRD is well recognized as a national representative data source for real-world evidence research and tends to draw its data from across the country thus minimising sampling bias [15].

The average age at diagnosis reported in the study (mean 42.68 years) which, although may be perceived as high, is in line with other recent UK studies. For example, Benoist et al. (2023)[16] report the mean age of 49 years for individuals with focal DRE in the UK. Other European countries reported similar findings: 51.4 years in Belgium, 50.7 years in Spain, 54.4 years in Italy, and 53.3 years in Germany, respectively [16]. For the overall study population, the mean age at focal DRE (F-DRE) diagnosis was 52.5 years as reported by Benoist et al. [16]. The authors also report that “*similar demographics were seen across the six participating countries. In the overall study cohort, the mean age at F-DRE diagnosis aligned with previously available evidence. In particular, a study of US veterans with DRE reported a mean age of 58.3. At the same time, a retrospective analysis of an Italian population found a mean age of 53 years for people with F-DRE* [16]*.*” The Benoist et al. study reports on focal epilepsy, whereas the current study was all-inclusive. The differences in the study population may explain that the average age at diagnosis was lower due to other epilepsy syndromes, for example. Another study by Hill et al. (2021) [17] also reports the mean age of patients on the date of validated diagnosis being 44.9 years. The authors postulate that this finding could be reflective of the protracted “treatment journeys” that people with epilepsy follow, including diagnosis and treatment delays which could lead to delays in diagnosis, and some patients could also have late-onset epilepsy [17].

In the study sample, 12.4% of people with epilepsy had intellectual disabilities. The figure was noticeably higher among children (23.5%). A greater proportion of younger patients in the subgroup of people with intellectual disabilities could contribute to the lower burden of comorbid conditions as measured by CCI and lower all-cause crude mortality compared to those without intellectual disabilities.

Of note is that the average CCI of 1.14 quoted with a large standard deviation (1.62) and a wide range (0–14) in our study, which is not compared to the other populations. It is to highlight that the current study is descriptive in nature, and not intended to therefore make any comparisons. However, a recent publication by Evans et al. (2023) [18] reported a mean CCI score of 1.0 with an SD of 1.5 due to disparities among people living with epilepsy in the UK. One reason for the wide deviation in the current study is due to the fact that we included both children and adults, as children typically have low CCI score.

In the current study, we found that the incidence and prevalence of DRE varied widely across different subgroups. People from socially deprived areas were over-represented in the study sample, which suggests that an enhanced targeted intervention for this population is needed. Consistent with the literature [19, 20], the incidence and prevalence of DRE were higher in people with epilepsy who had intellectual disabilities compared to those without intellectual disabilities. Females and children were also found to have a higher incidence and prevalence of DRE. Type of epilepsy also varied across subgroups. Further research is warranted to examine the relationship between type of epilepsy and DRE burden as well as clinical outcomes (e.g., mortality).

The findings showed that both the crude and standardised all-cause mortality rates were approximately four times higher in people with DRE compared to the general population in the UK during the same period. The trend was consistent over the study period. This finding highlights the need for early diagnosis and appropriate disease management for people with epilepsy who have an increased risk of DRE.

### Limitations and areas for further research

This study is not designed or scoped to compare between DRE, well-controlled people with epilepsy, and people without epilepsy. This study is based on the secondary use of electronic healthcare data in the CPRD databases, with data initially collected by clinicians in primary care settings. Therefore, the interpretation of data collected retrospectively is dependent on the completeness and quality of the medical records and the reliability of the abstraction of data from CPRD. Second, as a retrospective observational study, the current study is descriptive in nature and cannot establish causality between the baseline characteristics of people with DRE and post-index mortality. Given the descriptive nature of the study, relevant to all areas of the UK, covered by CRPD, it is also a limitation that further analysis was not undertaken on, for example, regional DRE prevalence or prevalence adjusted by age categories. This additional analysis is therefore an area for further research, which could include broadening the scope from a descriptive study to one that is more comparative in nature.

Third, the coding algorithms to define the study variables (e.g., DRE, intellectual disabilities) are yet to be validated using primary clinical records, which could affect the study results. The supplementary codes lists provided could be used in future studies to define these variables in CPRD and to assess the validity of the coding algorithms.

Fourth, although data from Wales are included in the CPRD datasets, such data are underrepresented [21, 22]. As a result, no patients from Wales were included in the study sample. Patient characteristics and treatment options might be different in Wales. Therefore, findings from the current study should be interpreted with caution when generalising results. In addition, the analysis of geographical distribution of DRE reflects the population coverage of CPRD practices, which does not necessarily align with the geographical distribution of DRE in the UK. Future studies with well-represented data are warranted to generate knowledge of DRE that can be generalised to all four countries in the UK.

Fifth, the follow-up period in the current study covered up to 10 years. However, the real-world patient journey may be much longer and complicated in the DRE population. Such patient journeys may include—but not be limited to—epilepsy diagnosis, ASM regimen change, DRE diagnosis, and additional diagnostic examinations such as electroencephalogram. As such, the study follow-up period may not capture the entire patient journey for those with DRE. Therefore, the incidence and prevalence of DRE may be underestimated in the current study.

Lastly, there was no minimum follow-up length required in the study. As such, people who died shortly after the index DRE diagnosis were included for analysis. However, patients with an index DRE date close to the end of the study may have a short follow-up that was insufficient for capturing disease outcomes.

## Conclusion

This study estimated the incidence and prevalence of DRE, characteristics, and mortality in a cohort of people with epilepsy from 2011 to 2021 based on data from CPRD in the UK and demonstrated considerable disease burden in people with DRE. Further research is warranted to examine the effect of patient characteristics on treatment pathway and clinical outcomes. The findings of the study may be generalised to people with epilepsy in the UK and thus may improve the understanding of disease burden associated with DRE and emphasise the importance of early diagnosis and appropriate disease management.

## Key message

- All-cause mortality was 4-fold higher among people with DRE compared to the general population in the UK.
-Great variations in DRE prevalence were observed across geographic locations and deprivation levels in the UK, which may have implication on quality of life and the need for resource allocation.
-Increased disease burden was observed in people with intellectual disabilities, indicating the need for personalised care in this population with DRE

### Supplementary Information

Below is the link to the electronic supplementary material.

Supplementary file1 (DOCX 84 KB)

## Data Availability

This study is based in part on data from the Clinical Practice Research Datalink obtained under licence from the UK Medicines and Healthcare Products Regulatory Agency. The data is provided by patients and collected by the NHS as part of their care and support. The interpretation and conclusions contained in this study are those of the author/s alone. Copyright^©^ 2023, re-used with the permission of The Health & Social Care Information Centre. All rights reserved.

## Abbreviations

ASM: Anti-seizure medication
CCI: Charlson comorbidity index
CI: (95%) Confidence interval
CPRD: Clinical practice research datalink
DEE: Developmental and epileptic encephalopathy
DRE: Drug-resistant epilepsy
F-DRE: Focal drug-resistant epilepsy
ILAE: International league against epilepsy
IMD: Index of multiple deprivation
IQR: Interquartile range
MHRA: Medicines and healthcare products regulatory agency
NHS: National Health Service
NIHR: National Institute for Health and Care Research
ONS: Office for National Statistics
RDG: Research Data Governance
SD: Standard deviation
SMR: Standardised mortality ratio
SQL: Structured Query Language
UK: United Kingdom
US: United States
