# Supplementary material for: Real-world evidence of epidemiology, patient characteristics, and mortality in people with drug-resistant epilepsy in the United Kingdom, 2011–2021
Source: J Neurol. 2024 Jan 19;271(5):2473–83. doi: 10.1007/s00415-023-12165-4 (PMC11055725; doi:10.1007/s00415-023-12165-4)
Supplement: Supplementary file 1 — Supplementary file1 (DOCX 84 KB) [file 415_2023_12165_MOESM1_ESM.docx]

# Supplementary Information (SI)

Journal of Neurology

Real-World Evidence of Epidemiology, Patient Characteristics, and Mortality in People with Drug-Resistant Epilepsy in the United Kingdom, 2011-2021

Rohit Shankar

Xiaocong L Marston*

Vanessa Danielson

Bronwyn Do Rego

Reginald Lasagne

Oliver Williams

Lara Groves

Corresponding Author: Xiaocong L Marston

Principal Consultant, Real World Evidence

OPEN Health, Evidence & Access

5 Churchill Place, 10th Floor London E14 5HU

[XiaocongLi@OpenHealthGroup.com](mailto:XiaocongLi@OpenHealthGroup.com)

## Online Resource Table 1. Code List for Epilepsy in CPRD

| Term | MedCodeId | Type of Epilepsy |
| --- | --- | --- |
| CPRD AURUM ^a^ | | |
| Complex partial seizure | 2564351000006115 | F |
| Witnessed epileptic seizure | 251269010 | U |
| Complex partial seizure with impairment of consciousness | 2564321000006112 | F |
| Epilepsy confirmed | 2159227017 | U |
| Atypical absence seizure | 2872771000006114 | G |
| Unverricht - Lundborg disease | 223781000000116 | DEE |
| Stress-induced epilepsy | 2474650018 | U |
| Generalized epilepsy | 2811081000006110 | G |
| O/E - grand mal fit | 254813013 | U |
| Epileptic seizures - clonic | 297282015 | U |
| Partial epilepsy with autonomic symptoms | 297312014 | F |
| Salaam attack | 2950441000006117 | U |
| [X]Other epilepsy | 299336018 | U |
| Epileptic seizures - atonic | 297266016 | G |
| Neonatal myoclonic epilepsy | 297281010 | G |
| Visceral reflex epilepsy | 297313016 | U |
| Cursive (running) epilepsy | 297325018 | F |
| Other forms of epilepsy NOS | 297330019 | U |
| Partial epilepsy with impairment of consciousness | 297295013 | F |
| Temporal lobe epilepsy | 297296014 | F |
| Epileptic automatism | 297302019 | U |
| Epileptic seizures - akinetic | 297267013 | U |
| Dysmnesic seizure | 2982351000006119 | F |
| Unilateral epilepsy | 297315011 | F |
| Other specified generalised convulsive epilepsy | 297289012 | G |
| Somatosensory epilepsy | 297311019 | F |
| Epileptic seizures - tonic | 297284019 | U |
| Partial epilepsy with impairment of consciousness NOS | 297303012 | F |
| Simple partial epileptic seizure | 301541000000116 | F |
| Psychosensory epilepsy | 297300010 | F |
| Other forms of epilepsy | 297324019 | U |
| [X]Other generalized epilepsy and epileptic syndromes | 299334015 | G |
| Epileptic seizures - myoclonic | 297283013 | G |
| Generalised convulsive epilepsy NOS | 297290015 | G |
| Infantile spasms | 297320011 | U |
| Post-seizure state | 3011621000006111 | U |
| Visual reflex epilepsy | 297314010 | U |
| Idiopathic generalised epilepsy | 3093151000006111 | G |
| Kojevnikov's epilepsy | 297321010 | F |
| Primary generalised epilepsy | 3093171000006118 | G |
| Atonic seizure | 3182921000006118 | G |
| Tonic seizure | 3341091000006114 | U |
| Severe myoclonic epilepsy in infancy | 345311015 | G |
| Menstrual epilepsy | 345321011 | U |
| Childhood absence epilepsy | 3323851000006114 | G |
| Pyridoxine-dependent epilepsy | 3506708010 | DEE |
| Nocturnal epilepsy | 345323014 | U |
| Focal epilepsy | 345225017 | F |
| Lennox-Gastaut syndrome | 345281011 | U |
| Simple partial seizure evolving to generalised seizure | 3498881000006114 | F |
| Tonic-clonic seizures | 3379251000006112 | U |
| Lafora disease | 345293019 | U |
| Ohtahara syndrome | 345302015 | DEE |
| Tonic-clonic seizure | 3379241000006110 | U |
| Symptomatic generalized epilepsy | 3668331000006116 | G |
| Drug-induced epilepsy | 345318014 | U |
| Juvenile absence epilepsy | 345275015 | G |
| Epileptic | 3878491000006111 | U |
| Simple partial seizure, consciousness not impaired | 3790311000006116 | F |
| Petit-mal seizure | 3795091000006119 | U |
| Epileptic disorder | 3878481000006113 | U |
| Reflex epilepsy | 3796891000006117 | U |
| Simple partial seizure with motor dysfunction | 3840421000006110 | F |
| EP - Epilepsy | 3878501000006115 | U |
| Sensory-induced epilepsy | 3796901000006118 | U |
| O/E - focal fit | 402501016 | U |
| Partial epilepsy | 245351000006115 | F |
| Progressive myoclonic epilepsy | 399395015 | G |
| O/E - focal (Jacksonian) fit | 402500015 | F |
| O/E - Jacksonian fit | 402502011 | F |
| Jacksonian, focal or motor epilepsy | 450603013 | F |
| Benign myoclonic epilepsy in infancy | 4769541000006119 | G |
| Tonic-clonic epilepsy | 472410017 | G |
| Myoclonic encephalopathy | 297060016 | DEE |
| Epilepsy NOS | 399408014 | U |
| Photogenic epilepsy | 4052921000006118 | U |
| Epileptic seizure | 453344019 | U |
| Somatosensory attacks | 3057051000006114 | F |
| Myoclonic seizures | 3101911000006116 | G |
| Infantile spasms | 46969011 | U |
| Grand mal epilepsy | 472411018 | U |
| TLE - Temporal lobe epilepsy | 4769611000006117 | F |
| Pykno-epilepsy | 495804010 | G |
| Myoclonic absence epilepsy | 5007661000006119 | G |
| Salaam attacks | 46971011 | U |
| Benign neonatal familial convulsions | 5007471000006111 | F |
| Motor epilepsy | 450602015 | U |
| Alcohol-induced epilepsy | 478024019 | U |
| Hypsarrhythmia | 46970012 | U |
| Lightning spasms | 46972016 | U |
| Mesiobasal limbic epilepsy | 4769641000006118 | F |
| Epilepsy undetermined whether focal or generalised | 5007871000006113 | U |
| West syndrome | 46968015 | U |
| Frontal lobe epilepsy | 5007261000006117 | F |
| Symptomatic myoclonic epilepsy | 5007811000006116 | F |
| Epilepsy with recurrent unilateral seizures in children | 5007131000006115 | F |
| Unverricht-Lundborg syndrome | 5007681000006112 | DEE |
| Sleep-related epilepsy | 5008031000006115 | U |
| Absence seizure | 504161016 | G |
| Simple partial onset of seizure with automatisms | 5231761000006118 | F |
| Absence seizure with atonic components | 5231901000006116 | G |
| Psychomotor epilepsy | 477355017 | F |
| Post-ictal state | 485041012 | U |
| Generalised convulsive epilepsy | 500033015 | G |
| Idiopathic myoclonic epilepsy | 5007781000006118 | G |
| Tonic-clonic convulsion | 3379261000006114 | U |
| Landau-Kleffner syndrome | 5007921000006115 | DEE |
| Photosensitive epilepsy | 512000013 | U |
| Absence seizure with tonic components | 5231911000006118 | G |
| Secondary generalized epilepsy | 3668341000006114 | F |
| Myoclonic seizure | 486701019 | U |
| Secondary reading epilepsy | 5007971000006119 | F |
| Occipital lobe epilepsy | 5007361000006110 | F |
| Epilepsy with grand mal seizures on awakening | 5007541000006119 | G |
| Isolated seizures | 5007841000006117 | U |
| Seizure with provoking factor | 5007861000006118 | U |
| Gelastic seizure | 3955151000006114 | F |
| Simple partial seizure with focal motor signs with march | 5231631000006118 | F |
| Epileptic fit | 6028641000006114 | U |
| Epileptic absences | 647301000006112 | G |
| Spasmus nutans | 6646631000006114 | U |
| Focal motor seizure | 4411881000006115 | F |
| Refractory infantile spasms | 6991361000006111 | DEE |
| Limbic system epilepsy | 743461000006119 | F |
| Intractable absence seizures | 8021441000006111 | G |
| Complex partial seizure of frontal lobe | 8024981000006113 | F |
| Absence seizure with impairment of consciousness only | 5231871000006116 | G |
| First generalized onset seizure | 8018431000006115 | U |
| Atypical absence epilepsy | 8053261000006111 | G |
| Epileptic encephalopathy | 7840671000006115 | DEE |
| Generalised nonconvulsive epilepsy NOS | 800721000006115 | G |
| First generalised onset seizure | 8018421000006118 | U |
| Refractory epilepsy | 7306821000006113 | U |
| Epilepsy co-occurrent and due to dementia | 7863681000006115 | U |
| Localization-related(focal)(partial)idiopathic epilepsy and epileptic syndromes with seizures of localised onset | 736371000006112 | F |
| Unverricht - Lundborg disease | 72751000006116 | DEE |
| Petit-mal epilepsy | 8191681000006117 | U |
| Infantile spasms -hysarrythmia | 883061000006119 | U |
| Petit mal epilepsy | 883041000006118 | U |
| Epilepsy associated problems | 918561000006111 | U |
| Nocturnal epilepsy | 918601000006111 | U |
| Partial complex seizure | 932141000006111 | F |
| Early infantile epileptic encephalopathy with suppression bursts | 931711000006118 | DEE |
| Juvenile myoclonic epilepsy | 11300012 | G |
| Tonic-clonic epilepsy | 103531000006110 | U |
| Night seizure | 1173961000000112 | U |
| Other specified generalised nonconvulsive epilepsy | 10791000006116 | G |
| Dravet Syndrome | 11778041000006119 | U |
| Gelastic epilepsy | 148427014 | F |
| Epilepsy | 178739011 | U |
| Many seizures a day | 2159222011 | U |
| Localisation-related epilepsy | 245341000006117 | F |
| Transient epileptic amnesia | 1489345013 | F |
| Dravet syndrome | 2193821000000118 | U |
| Generalised epilepsy | 223811000000118 | G |
| Petit mal (minor) epilepsy | 230321000006117 | U |
| O/E - psychomotor fit | 254819012 | U |
| Otohara syndrome | 253231000006116 | DEE |
| Sensory induced epilepsy | 148591000006114 | U |
| Grand mal seizure | 199568011 | U |
| Focal status epilepticus | 2502391000006115 | F |
| Epilepsia partialis continua | 2502351000006114 | F |
| Complex partial epileptic seizure | 2159274014 | F |
| Generalised non-convulsive epilepsy | 2550890017 | G |
| Epilepsy associated problems | 264634019 | U |
| O/E - petit mal fit | 254814019 | U |
| Partial epilepsy without impairment of consciousness OS | 245361000006118 | F |
| O/E - salaam attack | 254820018 | U |
| CPRD GOLD | | |
| Partial epilepsy with autonomic symptoms | 98870 | F |
| Epilepsy confirmed | 22341 | U |
| Simple partial epileptic seizure | 40105 | F |
| Neonatal myoclonic epilepsy | 37782 | G |
| Epilepsy associated problems | 50012 | O |
| Other specified partial epilepsy without mention of impairment of consciousness | 26733 | F |
| Seizures in response to acute event | 11505 | U |
| Somatosensory epilepsy | 37592 | F |
| Epilepsy | 573 | U |
| Petit mal (minor) epilepsy | 2907 | U |
| Myoclonic encephalopathy | 45602 | DEE |
| Other specified generalised convulsive epilepsy | 45927 | G |
| Complex partial epileptic seizure | 11394 | F |
| West syndrome | 39023 | U |
| Partial epilepsy without mention of impairment of consciousness NOS | 27526 | F |
| Partial epilepsy with impairment of consciousness NOS | 31920 | F |
| Transient epileptic amnesia | 38919 | F |
| Generalised nonconvulsive epilepsy | 11186 | G |
| O/E - grand mal fit | 7811 | U |
| Absence seizure | 8097 | G |
| O/E - petit mal fit | 7809 | U |
| Hypsarrhythmia | 7945 | U |
| Post-ictal state | 21885 | U |
| Dravet syndrome | 105679 | U |
| Many seizures a day | 39160 | U |
| Other specified generalised nonconvulsive epilepsy | 59185 | G |
| Temporal lobe epilepsy | 3175 | F |
| Tonic-clonic epilepsy | 8187 | U |
| Partial epilepsy with impairment of consciousness | 32288 | F |
| Other forms of epilepsy NOS | 9979 | U |
| Landau-Kleffner syndrome | 59806 | DEE |
| Focal epilepsy | 5525 | F |
| Epileptic seizures - tonic | 5152 | U |
| [X]Other epilepsy | 69831 | U |
| Gelastic epilepsy | 53483 | F |
| Unverricht - Lundborg disease | 63826 | DEE |
| Epileptic seizures - clonic | 18471 | U |
| Sensory induced epilepsy | 48134 | U |
| Ohtahara syndrome | 51998 | DEE |
| Visceral reflex epilepsy | 73542 | U |
| Lightning spasms | 68486 | U |
| Epilepsy NOS | 9747 | U |
| [X]Other generalized epilepsy and epileptic syndromes | 99731 | G |
| Epileptic seizures - akinetic | 31830 | U |
| Generalised convulsive epilepsy | 26144 | G |
| Unilateral epilepsy | 68946 | F |
| Spasmus nutans - nodding spasm | 21560 | U |
| Severe myoclonic epilepsy in infancy | 108409 | G |
| Generalised nonconvulsive epilepsy NOS | 44252 | G |
| Alcohol-induced epilepsy | 30604 | U |
| Grand mal seizure | 5668 | U |
| Motor epilepsy | 65699 | U |
| Epileptic automatism | 34079 | U |
| O/E - salaam attack | 31663 | U |
| Juvenile absence epilepsy | 17399 | G |
| Progressive myoclonic epilepsy | 37644 | G |
| Infantile spasms | 4478 | U |
| Fit (in known epileptic) NOS | 3607 | U |
| Nocturnal epilepsy | 4602 | U |
| Pykno-epilepsy | 99548 | G |
| [D]Nocturnal seizure | 99834 | U |
| Infantile spasms NOS | 49322 | U |
| Myoclonic seizure | 8487 | U |
| Cursive (running) epilepsy | 55260 | F |
| Lennox-Gastaut syndrome | 34792 | U |
| Partial epilepsy without mention of impairment of consciousness | 26015 | F |
| Drug-induced epilepsy | 30816 | U |
| Kojevnikov's epilepsy | 71719 | F |
| Early infantile epileptic encephalopathy with suppression bursts | 37906 | DEE |
| Localization-related(focal)(partial)idiopathic epilepsy and epileptic syndromes with seizures of localised onset | 9887 | F |
| Other forms of epilepsy | 38307 | U |
| O/E - Jacksonian fit | 39530 | F |
| Jacksonian, focal or motor epilepsy | 9569 | F |
| Limbic system epilepsy | 55665 | F |
| Epileptic seizures - myoclonic | 4801 | U |
| Juvenile myoclonic epilepsy | 19363 | G |
| Tonic-clonic epilepsy | 22804 | U |
| Salaam attacks | 23415 | U |
| Grand mal (major) epilepsy | 988 | U |
| Eye witness to epileptic seizure | 18378 | U |
| Menstrual epilepsy | 56359 | U |
| Epileptic absences | 1715 | G |
| O/E - focal (Jacksonian) fit | 57277 | U |
| O/E - focal fit | 12098 | U |
| Stress-induced epilepsy | 65673 | U |
| Epileptic seizures - atonic | 24309 | U |
| Otohara syndrome | 49340 | DEE |
| Generalised convulsive epilepsy NOS | 40806 | G |
| Photosensitive epilepsy | 30635 | G |
| Visual reflex epilepsy | 55739 | U |
| Psychomotor epilepsy | 23634 | F |
| Psychosensory epilepsy | 36203 | F |

Abbreviations: F, focal; G, generalised; DEE, developmental and epileptic encephalopathy; U, unclassifiable.

Note:

^a^ Clinical Practice Research Datalink (CPRD) is a database of de-identified coded primary care records for use in public health research. CPRD Aurum contains data contributed by general practices (GP) that use EMIS clinical systems, whilst CPRD GOLD contains data from a different GP software provider (InPS Vision). Due to differences in the structure and coding of the data between the two systems, the research databased have been released as separate data offerings (GOLD and Aurum). The medical codes [medcodeid] and the corresponding descriptions [term] are recorded medical conditions in CPRD.

## Online Resource Table 2. Code List for Anti-Seizure Medications (ASMs)

| BNF Chemical Name | BNF Chemical Code |
| --- | --- |
| Brivaracetam | 0408010AL |
| Carbamazepine | 0408010C0 |
| Clobazam | 040801060 |
| Clonazepam | 0408010F0 |
|  | 0408020D0 |
| Eslicarbazepine Acetate | 0408010AI |
| Ethosuximide | 0408010I0 |
| Felbamate | 0408010AA |
| Gabapentin | 0408010G0 |
| Lacosamide | 0408010AH |
| Lamotrigine | 0408010H0 |
| Levetiracetam | 0408010A0 |
| Midazolam | 0408020W0 |
|  | 0408020V0 |
| Oxcarbazepine | 0408010D0 |
| Paraldehyde | 0408020Q0 |
| Perampanel | 0408010AK |
| Phenobarbital | 0408010N0 |
|  | 0408010P0 |
| Phenytoin | 0408010Z0 |
| Phenytoin Sodium | 0408010Q0 |
|  | 0408020T0 |
| Pregabalin | 0408010AE |
| Primidone | 0408010U0 |
| Rufinamide | 0408010AF |
| Sodium Valproate | 0408010W0 |
| Stiripentol | 0408010AG |
| Sultiame | 0408010Y0 |
| Tiagabine | 0408010AB |
| Topiramate | 040801050 |
| Valproic Acid | 040801020 |
| Vigabatrin | 0408010X0 |
| Zonisamide | 0408010AD |

Abbreviation: BNF, British National Formulary.

## Online Resource Table 3. Study Variables and Operational Definitions

| Variable | Definition |
| --- | --- |
| DRE incidence and prevalence (Objective 1) | |
| Incidence | Incidence was estimated by dividing the number of new DRE cases in each calendar year by the total number of acceptable patients at risk with epilepsy enrolled in CPRD on 1 January of that year. For the last year (2021) when only partial data of the year were available (i.e., through 31 March), the incidence of the year was annualised by multiplying the number of new DRE cases in in the first 3 months of the year by 4. |
| Prevalence | The point-prevalence of DRE was estimated on 31 December of each calendar year by dividing the total number of people with DRE up to the date by the total number of people with epilepsy for each calendar year. |
| Patient characteristics and comorbidities (Objective 2) | |
| Age at index | Age in years: based on coded entry in CPRD, assuming a date of birth being 1 July of the birth year |
|  | Age by category: <18 years, 18 to 30 years, 31 to 40 years, 41 to 50 years, 51 to 60 years, 61 to 70 years, 71 years and above |
| Sex | Male and female: based on coded entry in CPRD |
| Index of Multiple Deprivation (IMD) | Most recent IMD data as recorded in CPRD was analysed by quintile from the most deprived 20% to the least deprived 20% (1 to 5) and a category for “unknown.” |
| Charlson Comorbidity Index (CCI) | CCI was derived using the Metcalfe methodology in CPRD [1] |
| Other comorbidities recorded in primary care | The top 10 most frequently recorded diagnoses were assessed based on the first 3 digits of the Read codes in CPRD within 1 year prior to the index DRE diagnosis |
| Time from epilepsy diagnosis to DRE diagnosis | The number of years between the first recorded epilepsy diagnosis and the index DRE diagnosis was assessed based on the corresponding dates in CPRD |
| Intellectual disabilities | People with and without a history of intellectual disabilities as recorded in CPRD |
| Geographic location | Most recent coded entry as recorded in CPRD: East Midlands, East of England, London, North East, North West, Scotland, South Central, South West, West Midlands, Yorkshire and the Humber |
| Type of epilepsy | The Wirrell et al. (2022) approach was adopted to define type of epilepsy using coded entry in CPRD from the first epilepsy diagnosis up to the day prior to the index DRE diagnosis [2]: generalised epilepsy, focal epilepsy, developmental and epileptic encephalopathy (DEE), focal and generalised epilepsy, generalised epilepsy and DEE, focal epilepsy and DEE, focal/generalised epilepsy and DEE, and unclassifiable epilepsy |
| Mortality (Objective 3) | |
| All-cause crude mortality | All-cause crude mortality was estimated by dividing the number of all deaths recorded in the ONS death registry in a given calendar year by the total number of alive people among those with DRE on 31 December of the year. For 2021, mortality was estimated on 31 March 2021. |
| Epilepsy-related crude mortality | Epilepsy-related crude mortality was estimated by dividing the number of people who were deceased with epilepsy as the primary cause of death as recorded in the ONS death registry in a given calendar year by the total number of alive people among those with DRE on 31 December of the year. For 2021, mortality was estimated on 31 March 2021. |
| Standardised all-cause mortality rate ratios (SMR) | All-cause SMR was estimated by dividing the age- and sex-adjusted observed all-cause mortality by the age- and sex-adjusted expected mortality in a reference European standardised population [3]. |
| Standardised epilepsy-related mortality rate ratios | Epilepsy-related was will be estimated by dividing the age- and sex-adjusted observed epilepsy-related mortality by the age- and sex-adjusted expected mortality in a reference European standardised population [3]. |

Abbreviations: CPRD, Clinical Practice Research Database; DRE, drug-resistant epilepsy; ONS, Office of National Statistics.

## Online Resource Table 4. Code List for Intellectual Disabilities

| Term | Read | Medcodeid |
| --- | --- | --- |
| CPRD Aurum ^a^ |  |  |
| Mental retardation | E3...00 | 151009017 |
| Mental subnormality NOS | E3...98 | 988941000006119 |
| Mental subnormality | E3...99 | 882761000006114 |
| Mild mental retardation, IQ in range 50-70 | E30..00 | 507246016 |
| Educationally subnormal | E30..11 | 413177014 |
| Feeble-minded | E30..12 | 667621000006119 |
| Moron | E30..13 | 215821000000119 |
| Mild mental retardation | E30..99 | 882771000006119 |
| Other specified mental retardation | E31..00 | 9881000006115 |
| Moderate mental retardation, IQ in range 35-49 | E310.00 | 700071000006118 |
| Imbecile | E310.11 | 785941000006115 |
| Moderate mental retardation | E310.99 | 882781000006116 |
| Severe mental retardation, IQ in range 20-34 | E311.00 | 146051000006113 |
| Severe mental retardation | E311.99 | 882791000006118 |
| Profound mental retardation with IQ less than 20 | E312.00 | 201751000006110 |
| Idiocy | E312.11 | 787151000006113 |
| Other specified mental retardation NOS | E31z.00 | 295662012 |
| Mental subnormality NOS | E31z.99 | 882801000006117 |
| Other specified mental retardation | E3y..00 | 295661017 |
| Intellectual disability | E3z..00 | 295664013 |
| [X]Mental retardation | Eu7..00 | 398231000006111 |
| [X]Mild mental retardation | Eu70.00 | 398381000006119 |
| [X]Feeble-mindedness | Eu70.11 | 386751000006111 |
| [X]Mild mental subnormality | Eu70.12 | 398411000006116 |
| [X]Mld mental retard with statement no or min impairm behav | Eu70000 | 398661000006116 |
| [X]Mld mental retard sig impairment behav req attent/treatmt | Eu70100 | 398651000006118 |
| [X]Mild mental retardation, other impairments of behaviour | Eu70y00 | 296557014 |
| Mild intellectual disability | Eu70z00 | 398391000006116 |
| Moderate intellectual disability | Eu71.00 | 398811000006118 |
| [X]Moderate mental subnormality | Eu71.11 | 398821000006114 |
| [X]Mod mental retard with statement no or min impairm behav | Eu71000 | 398771000006118 |
| [X]Mod mental retard sig impairment behav req attent/treatmt | Eu71100 | 398761000006113 |
| [X]Mod retard oth behav impair | Eu71y00 | 296565012 |
| [X]Mod mental retardation without mention impairment behav | Eu71z00 | 398781000006115 |
| Severe intellectual disability | Eu72.00 | 426591000006111 |
| [X]Severe mental subnormality | Eu72.11 | 426611000006117 |
| [X]Sev mental retard with statement no or min impairm behav | Eu72000 | 426531000006112 |
| [X]Sev mental retard sig impairment behav req attent/treatmt | Eu72100 | 426521000006114 |
| [X]Severe mental retardation, other impairments of behaviour | Eu72y00 | 296574014 |
| [X]Sev mental retardation without mention impairment behav | Eu72z00 | 426541000006119 |
| Profound intellectual disability | Eu73.00 | 423501000006112 |
| [X]Profound mental subnormality | Eu73.11 | 423521000006119 |
| [X]Profound ment retrd wth statement no or min impairm behav | Eu73000 | 423491000006116 |
| [X]Profound ment retard sig impairmnt behav req attent/treat | Eu73100 | 423481000006119 |
| [X]Profound mental retardation, other impairments of behavr | Eu73y00 | 423511000006110 |
| [X]Prfnd mental retardation without mention impairment behav | Eu73z00 | 423361000006118 |
| [X]Other mental retardation | Eu7y.00 | 296586012 |
| [X]Oth mental retard with statement no or min impairm behav | Eu7y000 | 404861000006115 |
| [X]Oth mental retard sig impairment behav req attent/treatmt | Eu7y100 | 404851000006117 |
| [X]Other mental retardation, other impairments of behaviour | Eu7yy00 | 296592018 |
| [X]Other mental retardation without mention impairment behav | Eu7yz00 | 411791000006118 |
| [X]Unspecified mental retardation | Eu7z.00 | 401902015 |
| [X]Mental deficiency NOS | Eu7z.11 | 398201000006115 |
| [X]Mental subnormality NOS | Eu7z.12 | 398251000006116 |
| [X]Unsp mental retard with statement no or min impairm behav | Eu7z000 | 430061000006113 |
| [X]Unsp mentl retard sig impairment behav req attent/treatmt | Eu7z100 | 430081000006115 |
| [X]Unspecified mental retardatn, other impairments of behav | Eu7zy00 | 431231000006115 |
| [X]Unsp mental retardation without mention impairment behav | Eu7zz00 | 430071000006118 |
| [X]Disorders of psychological development | Eu8..00 | 377921000006114 |
| [X]Specific developmental disorders of speech and language | Eu80.00 | 427261000006119 |
| [X]Specific speech articulation disorder | Eu80000 | 427311000006114 |
| Developmental speech articulation disorder | Eu80011 | 376961000006117 |
| [X]Developmental speech articulation disorder | Eu80012 | 1491711019 |
| [X]Dyslalia | Eu80013 | 1495372013 |
| [X]Functional speech articulation disorder | Eu80014 | 1495048014 |
| [X]Lalling | Eu80015 | 1495484014 |
| Expressive language disorder | Eu80100 | 384371000006112 |
| Developmental expressive language disorder | Eu80111 | 376911000006115 |
| [X]Developmental aphasia, expressive type | Eu80112 | 376841000006114 |
| Receptive language disorder | Eu80200 | 401905018 |
| [X]Congenital auditory imperception | Eu80211 | 371571000006114 |
| [X]Developmental dysphasia, receptive type | Eu80212 | 376921000006111 |
| [X]Developmental Wernicke's aphasia | Eu80213 | 376981000006110 |
| [X]Word deafness | Eu80214 | 432821000006113 |
| Receptive language delay | Eu80215 | 376851000006111 |
| Acquired epileptic aphasia | Eu80300 | 362161000006110 |
| [X]Cocktail party syndrome | Eu80400 | 301521000000111 |
| [X]Semantic-pragmatic disorder | Eu80500 | 556671000000113 |
| Auditory processing disorder | Eu80600 | 1770581000006110 |
| Acquired receptive language impairment | Eu80700 | 1947961000006110 |
| Developmental receptive language disorder | Eu80800 | 1947971000006110 |
| Acquired language comprehension impairment | Eu80900 | 1947981000006110 |
| Developmental language comprehension impairment | Eu80A00 | 1947991000006110 |
| Speech disorder | Eu80B00 | 1948001000006110 |
| [X]Other developmental disorders of speech and language | Eu80y00 | 401906017 |
| [X]Lisping | Eu80y11 | 395241000006114 |
| [X]Developmental disorder of speech and language unspecified | Eu80z00 | 376891000006117 |
| [X]Language development disorder NOS | Eu80z11 | 394991000006113 |
| [X]Specific developmental disorders of scholastic skills | Eu81.00 | 296643017 |
| [X]Specific reading disorder | Eu81000 | 427291000006110 |
| [X]Backward reading | Eu81011 | 366631000006110 |
| [X]Developmental dyslexia | Eu81012 | 376901000006118 |
| [X]Specific reading retardation | Eu81013 | 427301000006111 |
| [X]Specific spelling disorder | Eu81100 | 427321000006118 |
| [X]Specific spelling retardation without reading disorder | Eu81111 | 427331000006115 |
| [X]Specific disorder of arithmetical skills | Eu81200 | 296651019 |
| [X]Developmental acalculia | Eu81211 | 376821000006119 |
| [X]Developmental arithmetical disorder | Eu81212 | 376861000006113 |
| [X]Developmental Gerstmann's syndrome | Eu81213 | 376951000006119 |
| Mixed disorder of scholastic skills | Eu81300 | 398591000006113 |
| Moderate learning disability | Eu81400 | 1129811000000110 |
| Severe learning disability | Eu81500 | 1129781000000110 |
| Mild learning disability | Eu81600 | 1550041000000110 |
| Profound learning disability | Eu81700 | 1550051000000110 |
| Specific learning disability | Eu81800 | 1887331000006110 |
| [X]Other developmental disorders of scholastic skills | Eu81y00 | 401909012 |
| [X]Developmental expressive writing disorder | Eu81y11 | 376941000006116 |
| Developmental disorder of scholastic skill | Eu81z00 | 296657015 |
| Learning disability | Eu81z11 | 395061000006117 |
| [X]Learning disorder NOS | Eu81z12 | 395071000006112 |
| [X]Learn acquisition disab NOS | Eu81z13 | 395051000006119 |
| [X]Specific developmental disorder of motor function | Eu82.00 | 1495285013 |
| [X]Clumsy child syndrome | Eu82.11 | 1490642014 |
| [X]Developmental co - ordination disorder | Eu82.12 | 1495322011 |
| [X]Developmental dyspraxia | Eu82.13 | 376931000006114 |
| Mixed developmental disorder | Eu83.00 | 296663012 |
| Autism spectrum disorder | Eu84.00 | 420581000006111 |
| [X]Childhood autism | Eu84000 | 370801000006114 |
| Autistic disorder | Eu84011 | 366581000006112 |
| [X]Infantile autism | Eu84012 | 389671000006119 |
| [X]Infantile psychosis | Eu84013 | 389681000006116 |
| [X]Kanner's syndrome | Eu84014 | 394581000006118 |
| Atypical autism | Eu84100 | 366531000006111 |
| [X]Atypical childhood psychosis | Eu84111 | 366551000006116 |
| [X]Mental retardation with autistic features | Eu84112 | 398241000006118 |
| Rett syndrome | Eu84200 | 424971000006111 |
| [X]Other childhood disintegrative disorder | Eu84300 | 401910019 |
| [X]Dementia infantalis | Eu84311 | 376631000006115 |
| [X]Disintegrative psychosis | Eu84312 | 377461000006113 |
| [X]Heller's syndrome | Eu84313 | 388761000006114 |
| [X]Symbiotic psychosis | Eu84314 | 428441000006116 |
| [X]Overactive disorder assoc mental retard/stereotype movts | Eu84400 | 417681000006116 |
| Asperger's syndrome | Eu84500 | 363931000006110 |
| [X]Autistic psychopathy | Eu84511 | 366591000006110 |
| [X]Schizoid disorder of childhood | Eu84512 | 425581000006116 |
| [X]Other pervasive developmental disorders | Eu84y00 | 296678017 |
| [X]Pervasive developmental disorder, unspecified | Eu84z00 | 296679013 |
| Autistic spectrum disorder | Eu84z11 | 2535850012 |
| [X]Global developmental delay | Eu85.00 | 1667721000000110 |
| [X]Neurodevelopmental delay | Eu86.00 | 1667711000000110 |
| [X]Other disorders of psychological development | Eu8y.00 | 401911015 |
| [X]Developmental agnosia | Eu8y.11 | 376831000006116 |
| Disorder of psychological development | Eu8z.00 | 296683013 |
| [X]Psychological developmental disorder NOS | Eu8z.11 | 424181000006111 |
| CPRD GOLD |  |  |
| Mental retardation | E3...00 | 1362 |
| Mild mental retardation, IQ in range 50-70 | E30..00 | 1787 |
| Educationally subnormal | E30..11 | 1680 |
| Feeble-minded | E30..12 | 51954 |
| Moron | E30..13 | 71632 |
| Other specified mental retardation | E31..00 | 56577 |
| Moderate mental retardation, IQ in range 35-49 | E310.00 | 302 |
| Imbecile | E310.11 | 1278 |
| Severe mental retardation, IQ in range 20-34 | E311.00 | 4825 |
| Profound mental retardation with IQ less than 20 | E312.00 | 45133 |
| Idiocy | E312.11 | 51622 |
| Other specified mental retardation NOS | E31z.00 | 54179 |
| Other specified mental retardation | E3y..00 | 57199 |
| Mental retardation NOS | E3z..00 | 37867 |
| [X]Mental retardation | Eu7..00 | 28962 |
| [X]Mild mental retardation | Eu70.00 | 28740 |
| [X]Feeble-mindedness | Eu70.11 | 84154 |
| [X]Mild mental subnormality | Eu70.12 | 33949 |
| [X]Mld mental retard with statement no or min impairm behav | Eu70000 | 46504 |
| [X]Mld mental retard sig impairment behav req attent/treatmt | Eu70100 | 39412 |
| [X]Mild mental retardation, other impairments of behaviour | Eu70y00 | 39016 |
| [X]Mild mental retardation without mention impairment behav | Eu70z00 | 50606 |
| [X]Moderate mental retardation | Eu71.00 | 6123 |
| [X]Moderate mental subnormality | Eu71.11 | 34734 |
| [X]Mod mental retard with statement no or min impairm behav | Eu71000 | 60913 |
| [X]Mod mental retard sig impairment behav req attent/treatmt | Eu71100 | 54881 |
| [X]Mod retard oth behav impair | Eu71y00 | 59407 |
| [X]Mod mental retardation without mention impairment behav | Eu71z00 | 60473 |
| [X]Severe mental retardation | Eu72.00 | 36143 |
| [X]Severe mental subnormality | Eu72.11 | 27691 |
| [X]Sev mental retard with statement no or min impairm behav | Eu72000 | 50947 |
| [X]Sev mental retard sig impairment behav req attent/treatmt | Eu72100 | 50751 |
| [X]Severe mental retardation, other impairments of behaviour | Eu72y00 | 55848 |
| [X]Sev mental retardation without mention impairment behav | Eu72z00 | 55560 |
| [X]Profound mental retardation | Eu73.00 | 51268 |
| [X]Profound mental subnormality | Eu73.11 | 65468 |
| [X]Profound ment retrd wth statement no or min impairm behav | Eu73000 | 70102 |
| [X]Profound ment retard sig impairmnt behav req attent/treat | Eu73100 | 98100 |
| [X]Profound mental retardation, other impairments of behavr | Eu73y00 | 90276 |
| [X]Prfnd mental retardation without mention impairment behav | Eu73z00 | 60062 |
| [X]Other mental retardation | Eu7y.00 | 71196 |
| [X]Oth mental retard with statement no or min impairm behav | Eu7y000 | 70008 |
| [X]Oth mental retard sig impairment behav req attent/treatmt | Eu7y100 | 56547 |
| [X]Other mental retardation, other impairments of behaviour | Eu7yy00 | 42520 |
| [X]Other mental retardation without mention impairment behav | Eu7yz00 | 63273 |
| [X]Unspecified mental retardation | Eu7z.00 | 42589 |
| [X]Mental deficiency NOS | Eu7z.11 | 37887 |
| [X]Mental subnormality NOS | Eu7z.12 | 37911 |
| [X]Unsp mental retard with statement no or min impairm behav | Eu7z000 | 42886 |
| [X]Unsp mentl retard sig impairment behav req attent/treatmt | Eu7z100 | 66783 |
| [X]Unspecified mental retardatn, other impairments of behav | Eu7zy00 | 66383 |
| [X]Unsp mental retardation without mention impairment behav | Eu7zz00 | 32820 |
| [X]Disorders of psychological development | Eu8..00 | 25205 |
| [X]Specific developmental disorders of speech and language | Eu80.00 | 6122 |
| [X]Specific speech articulation disorder | Eu80000 | 31813 |
| [X]Developmental phonological disorder | Eu80011 | 8473 |
| [X]Developmental speech articulation disorder | Eu80012 | 40364 |
| [X]Dyslalia | Eu80013 | 98764 |
| [X]Functional speech articulation disorder | Eu80014 | 62010 |
| [X]Expressive language disorder | Eu80100 | 9594 |
| [X]Developmental dysphasia, expressive type | Eu80111 | 7139 |
| [X]Developmental aphasia, expressive type | Eu80112 | 46236 |
| [X]Receptive language disorder | Eu80200 | 30449 |
| [X]Congenital auditory imperception | Eu80211 | 64488 |
| [X]Developmental dysphasia, receptive type | Eu80212 | 53844 |
| [X]Developmental Wernicke's aphasia | Eu80213 | 112349 |
| [X]Word deafness | Eu80214 | 113830 |
| [X]Developmental aphasia, receptive type | Eu80215 | 56772 |
| [X]Acquired aphasia with epilepsy [Landau - Kleffner] | Eu80300 | 43679 |
| [X]Cocktail party syndrome | Eu80400 | 65373 |
| [X]Semantic-pragmatic disorder | Eu80500 | 94166 |
| [X]Auditory processing disorder | Eu80600 | 102890 |
| [X]Acquired receptive language impairment | Eu80700 | 115004 |
| [X]Developmental receptive language impairment | Eu80800 | 114639 |
| [X]Speech disorder | Eu80B00 | 109738 |
| [X]Other developmental disorders of speech and language | Eu80y00 | 46054 |
| [X]Lisping | Eu80y11 | 62892 |
| [X]Developmental disorder of speech and language unspecified | Eu80z00 | 6514 |
| [X]Language development disorder NOS | Eu80z11 | 73932 |
| [X]Specific developmental disorders of scholastic skills | Eu81.00 | 21214 |
| [X]Specific reading disorder | Eu81000 | 28330 |
| [X]Backward reading | Eu81011 | 24104 |
| [X]Developmental dyslexia | Eu81012 | 24762 |
| [X]Specific reading retardation | Eu81013 | 55406 |
| [X]Specific spelling disorder | Eu81100 | 72498 |
| [X]Specific spelling retardation without reading disorder | Eu81111 | 68900 |
| [X]Specific disorder of arithmetical skills | Eu81200 | 62766 |
| [X]Developmental acalculia | Eu81211 | 68753 |
| [X]Developmental Gerstmann's syndrome | Eu81213 | 57162 |
| [X]Mixed disorder of scholastic skills | Eu81300 | 43798 |
| [X]Moderate learning disability | Eu81400 | 98342 |
| [X]Severe learning disability | Eu81500 | 98293 |
| [X]Mild learning disability | Eu81600 | 99774 |
| [X]Profound learning disability | Eu81700 | 100648 |
| [X]Specific learning disability | Eu81800 | 107968 |
| [X]Other developmental disorders of scholastic skills | Eu81y00 | 44342 |
| [X]Developmental expressive writing disorder | Eu81y11 | 23976 |
| [X]Developmental disorder of scholastic skills, unspecified | Eu81z00 | 6516 |
| [X]Learning disability NOS | Eu81z11 | 4477 |
| [X]Learning disorder NOS | Eu81z12 | 16855 |
| [X]Learn acquisition disab NOS | Eu81z13 | 36045 |
| [X]Specific developmental disorder of motor function | Eu82.00 | 7299 |
| [X]Clumsy child syndrome | Eu82.11 | 20518 |
| [X]Developmental co - ordination disorder | Eu82.12 | 37763 |
| [X]Developmental dyspraxia | Eu82.13 | 21062 |
| [X]Mixed specific developmental disorders | Eu83.00 | 6513 |
| [X]Pervasive developmental disorders | Eu84.00 | 7226 |
| [X]Childhood autism | Eu84000 | 3637 |
| [X]Autistic disorder | Eu84011 | 9982 |
| [X]Infantile autism | Eu84012 | 50337 |
| [X]Infantile psychosis | Eu84013 | 61304 |
| [X]Kanner's syndrome | Eu84014 | 110478 |
| [X]Atypical autism | Eu84100 | 24044 |
| [X]Atypical childhood psychosis | Eu84111 | 24062 |
| [X]Mental retardation with autistic features | Eu84112 | 34174 |
| [X]Rett's syndrome | Eu84200 | 31042 |
| [X]Other childhood disintegrative disorder | Eu84300 | 68299 |
| [X]Dementia infantalis | Eu84311 | 101999 |
| [X]Disintegrative psychosis | Eu84312 | 62222 |
| [X]Heller's syndrome | Eu84313 | 46429 |
| [X]Symbiotic psychosis | Eu84314 | 53848 |
| [X]Overactive disorder assoc mental retard/stereotype movts | Eu84400 | 52602 |
| [X]Asperger's syndrome | Eu84500 | 2950 |
| [X]Autistic psychopathy | Eu84511 | 51375 |
| [X]Schizoid disorder of childhood | Eu84512 | 59285 |
|  | Eu84600 | 111828 |
| [X]Other pervasive developmental disorders | Eu84y00 | 47948 |
| [X]Pervasive developmental disorder, unspecified | Eu84z00 | 44327 |
| [X]Autistic spectrum disorder | Eu84z11 | 42941 |
| [X]Global developmental delay | Eu85.00 | 100899 |
| [X]Neurodevelopmental delay | Eu86.00 | 101187 |
| [X]Other disorders of psychological development | Eu8y.00 | 44680 |
| [X]Unspecified disorder of psychological development | Eu8z.00 | 56981 |
| [X]Psychological developmental disorder NOS | Eu8z.11 | 107682 |
| Language-related cognitive disorder | ZS3..00 | 40002 |
| Dysgraphia | ZS31.00 | 39428 |
| Deep dysgraphia | ZS31100 | 42027 |
| Phonological dysgraphia | ZS31200 | 85496 |
| Paragraphia | ZS31400 | 113788 |
| Dyslexia | ZS32.00 | 10265 |
| Specific reading difficulty | ZS32.11 | 57256 |
| Specific reading retardation | ZS32.12 | 66827 |
| Deep dyslexia | ZS32100 | 65510 |
| Phonological dyslexia | ZS32200 | 55891 |
| Surface dyslexia | ZS32300 | 72563 |
| Agraphia | ZS33.00 | 70820 |
| Developmental disorder of scholastic skill | ZS34.00 | 39128 |
| Learning disability | ZS34.11 | 19436 |
| Specific spelling disorder | ZS34100 | 110115 |
| Speech and language dyspraxias | ZS5..00 | 43892 |
| Apraxia of speech | ZS51.00 | 47008 |
| Oral dyspraxia | ZS52.00 | 48273 |
| Oral apraxia | ZS52.11 | 98834 |
| Verbal dyspraxia | ZS53.00 | 32326 |
| Verbal apraxia | ZS53.11 | 104383 |
| Developmental verbal dyspraxia | ZS53100 | 22164 |
| DVD - Developmental verbal apraxia | ZS53111 | 44797 |
| Oral-verbal dyspraxia | ZS54.00 | 65462 |
| Articulatory dyspraxia | ZS55.00 | 54303 |
| Developmental articulatory dyspraxia | ZS55100 | 56004 |
| DAD - Developmental articulatory dyspraxia | ZS55111 | 37970 |
| Immature articulatory praxis | ZS56.00 | 68812 |
| IAP - Immature articulatory praxis | ZS56.11 | 113943 |
| Dyspraxia of velopharynx | ZS57.00 | 50159 |
| Tongue tip dyspraxia | ZS59.00 | 63181 |
| Speech and phonology impairments | ZS6..00 | 28845 |
| Phonological impairment | ZS61.00 | 52438 |
| Phonology impairment | ZS61.11 | 67917 |
| Sound system impairment | ZS61.12 | 56597 |
| Articulation impairment | ZS61.13 | 58534 |
| Articulation disorder | ZS61.14 | 62451 |
| Phonological programming deficit | ZS62.00 | 105658 |
| Restricted sound system | ZS63.00 | 71738 |
| Limited sound system | ZS64.00 | 68124 |
| Phonological disorder | ZS66.00 | 40067 |
| Abnormal phonology | ZS66.11 | 69989 |
| Deviant phonology | ZS66.12 | 59668 |
| Phonology disorder | ZS66.13 | 40051 |
| Speech impairment | ZS67.00 | 18452 |
| Developmental speech disorder | ZS67100 | 25574 |
| DSD - Developmental speech disorder | ZS67111 | 39144 |
| Phonological delay | ZS67200 | 30467 |
| Phonology delay | ZS67211 | 55245 |
| Speech delay | ZS67300 | 11420 |
| Slow to talk | ZS67311 | 46922 |
| Immature sound system | ZS67400 | 68459 |
| Language impairment | ZS7..00 | 40065 |
| Expressive language impairment | ZS71.00 | 63886 |
| Receptive language impairment | ZS72.00 | 32542 |
| Specific language impairment | ZS73.00 | 59178 |
| Specific language disorder | ZS73.11 | 41238 |
| Lexical syntactic disorder | ZS74.00 | 94715 |
| Semantic-pragmatic disorder | ZS75.00 | 42799 |
| Auditory processing disorder | ZS76.00 | 51200 |
| Dysphasia | ZS78.00 | 9992 |
| Aphasia | ZS78.11 | 11598 |
| Acquired dysphasias | ZS78100 | 48292 |
| Mixed transcortical dysphasia | ZS78200 | 64964 |
| Isolation aphasia | ZS78211 | 112366 |
| Isolation dysphasia | ZS78212 | 95596 |
| Subcortical aphasia | ZS78300 | 98539 |
| Transcortical motor dysphasia | ZS78400 | 112862 |
| Transcortical motor aphasia | ZS78411 | 93178 |
| Anomia | ZS78500 | 26170 |
| Anomic aphasia | ZS78511 | 37771 |
| Anomic dysphasia | ZS78512 | 56551 |
| Nominal dysphasia | ZS78513 | 35192 |
| Transcortical sensory dysphasia | ZS78600 | 101822 |
| Transcortical sensory aphasia | ZS78611 | 105929 |
| Frontal dynamic dysphasia | ZS78700 | 107238 |
| Global dysphasia | ZS78800 | 50602 |
| Global aphasia | ZS78811 | 63425 |
| Mixed dysphasia | ZS78900 | 52686 |
| Mixed aphasia | ZS78911 | 68774 |
| Semantic dysphasia | ZS78A00 | 89004 |
| Semantic aphasia | ZS78A11 | 113917 |
| Conduction dysphasia | ZS78B00 | 113777 |
| Conduction aphasia | ZS78B11 | 93486 |
| Receptive dysphasia | ZS78C00 | 49609 |
| Receptive aphasia | ZS78C11 | 57542 |
| Wernicke's dysphasia | ZS78D00 | 65074 |
| Jargon aphasia | ZS78D11 | 101320 |
| Jargon dysphasia | ZS78D12 | 66978 |
| Wernicke's aphasia | ZS78D13 | 51158 |
| Fluent dysphasia | ZS78E00 | 64191 |
| Fluent aphasia | ZS78E11 | 67135 |
| Posterior dysphasia | ZS78F00 | 98050 |
| Expressive dysphasia | ZS78G00 | 10110 |
| Expressive aphasia | ZS78G11 | 28872 |
| Broca's dysphasia | ZS78H00 | 48919 |
| Broca's aphasia | ZS78H11 | 54017 |
| Non-fluent dysphasia | ZS78I00 | 50803 |
| Non-fluent aphasia | ZS78I11 | 91135 |
| Efferent motor dysphasia | ZS78K00 | 71374 |
| Efferent motor aphasia | ZS78K11 | 67588 |
| Developmental dysphasia | ZS78L00 | 35238 |
| Syntactic difficulties | ZS79.11 | 106612 |
| Syntactic disorder | ZS79.12 | 105545 |
| Developmental syntactic impairment | ZS79100 | 90873 |
| Developmental syntactic difficulties | ZS79111 | 22420 |
| Developmental syntactic disorder | ZS79112 | 99610 |
| Semantic impairment | ZS7A.00 | 64372 |
| Semantic deficit | ZS7A.11 | 61058 |
| Developmental semantic impairment | ZS7A100 | 70285 |
| Developmental semantic disorder | ZS7A111 | 61312 |
| Semantic-pragmatic impairment | ZS7A200 | 57354 |
| Developmental language impairment | ZS7B.00 | 45578 |
| Expressive language delay | ZS7B100 | 12372 |
| Receptive language delay | ZS7B200 | 24955 |
| Word finding difficulty | ZS7B300 | 33998 |
| Developmental language delay | ZS7B400 | 9522 |
| Developmental language disorder | ZS7B500 | 10345 |
| Delayed pre-verbal development | ZS7B600 | 42202 |
| Restricted language development | ZS7B700 | 54141 |
| Restricted expressive language development | ZS7B800 | 62139 |
| Restricted receptive language development | ZS7B900 | 90194 |
| Congenital auditory imperception | ZS7BA00 | 109453 |
| Discourse difficulties | ZS7BB00 | 48504 |
| Acquired language disorder | ZS7C.00 | 47090 |
| Grapheme-phoneme conversion deficit | ZS7C200 | 50352 |
| Language disorder associated with right hemisphere damage | ZS7C400 | 99857 |
| Language disorder of dementia | ZS7C500 | 55222 |
| Language disorder associated with thought disorder | ZS7C600 | 32831 |
| Schizophrenic language | ZS7C611 | 57376 |
| Post-traumatic mutism | ZS7C700 | 99609 |
| Sociolinguistic difficulties | ZS7D.00 | 30553 |
| Disorder of speech and language development | ZS8..00 | 25102 |
| Developmental expressive language disorder | ZS81.00 | 45472 |
| Acquired epileptic aphasia | ZS82.00 | 49889 |
| Landau-Kleffner syndrome | ZS82.11 | 59806 |
| Developmental speech articulation disorder | ZS83.00 | 66828 |
| Expressive language disorder | ZS84.00 | 32305 |
| Disorders of attention and motor control | ZS9..00 | 37994 |
| Attention deficit disorder | ZS91.00 | 28543 |
| ADD - Attention deficit disorder | ZS91.11 | 24808 |
| [X]Attention deficit disorder | ZS91.12 | 24753 |
| Persistent developmental avoidance | ZS92.00 | 59683 |
| PDA - Persistent developmental avoidance | ZS92.11 | 25376 |
| Deficits in attention motor control and perception | ZS93.00 | 39920 |
| DAMP - Deficits in attention motor control and perception | ZS93.11 | 35161 |
| Minimal brain dysfunction | ZS94.00 | 37894 |
| MBD - Minimal brain dysfunction | ZS94.11 | 61701 |
| Soft neurological signs | ZS94.12 | 50129 |

Note:

^a^ Clinical Practice Research Datalink (CPRD) is a database of de-identified coded primary care records for use in public health research. CPRD Aurum contains data contributed by general practices (GP) that use EMIS clinical systems, whilst CPRD GOLD contains data from a different GP software provider (InPS Vision). Due to differences in the structure and coding of the data between the two systems, the research databases have been released as separate data offerings (GOLD and Aurum). The medical codes [Read and Medcodeid] and the corresponding descriptions [Term] are recorded medical conditions in CPRD.

References

1. Metcalfe D, Masters J, Delmestri A, Judge A, Perry D, Zogg C et al (2019) Coding algorithms for defining Charlson and Elixhauser co-morbidities in Read-coded databases*.* BMC medical research methodology 19(1): 1-9 <https://doi.org/10.1186/s12874-019-0753-5>

2. Wirrell EC, Nabbout R, Scheffer IE, Alsaadi T, Bogacz A, French JA et al (2022) Methodology for classification and definition of epilepsy syndromes with list of syndromes: report of the ILAE Task Force on Nosology and Definitions*.* Epilepsia 63(6): 1333-1348 <https://doi.org/10.1111/epi.17237>

3. Deaths registered by area of usual residence, UK. Office for National Statistics (ONS) 01 December 2021. <https://www.ons.gov.uk/peoplepopulationandcommunity/birthsdeathsandmarriages/deaths/datasets/deathsregisteredbyareaofusualresidenceenglandandwales>. Accessed 27 July 2023
